# Supplementary figures and images for: RNA-protein binding motifs mining with a new hybrid deep learning based cross-domain knowledge integration approach
Source: BMC Bioinformatics. 2017 Feb 28;18:136. doi: 10.1186/s12859-017-1561-8 (PMC5331642; doi:10.1186/s12859-017-1561-8)

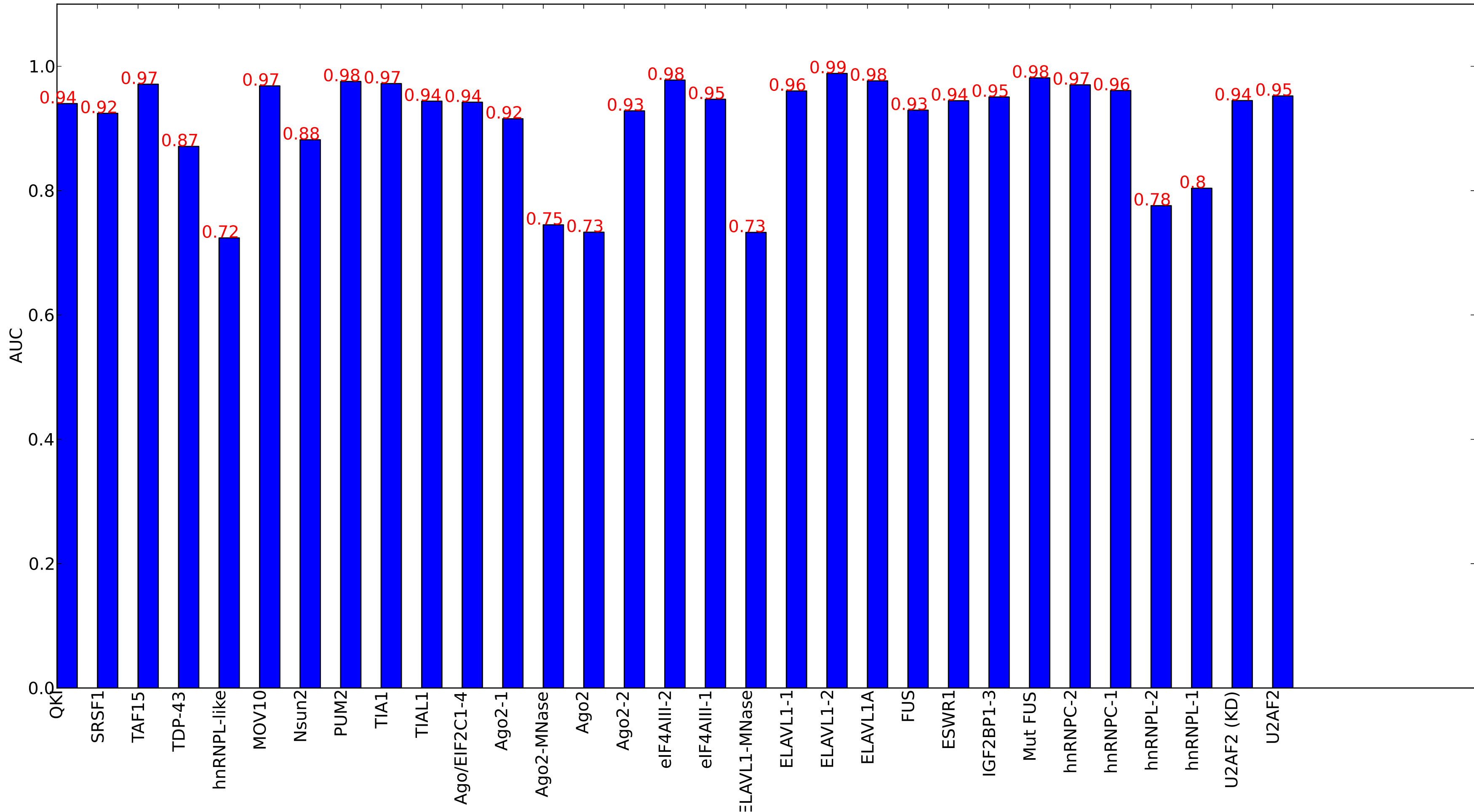

Supplement: Additional file 1 — Figure S1. The AUCs of 5-fold cross-validation across 31 experiment datasets using iDeep. (PDF 24 kb) [file 12859_2017_1561_MOESM1_ESM.pdf]

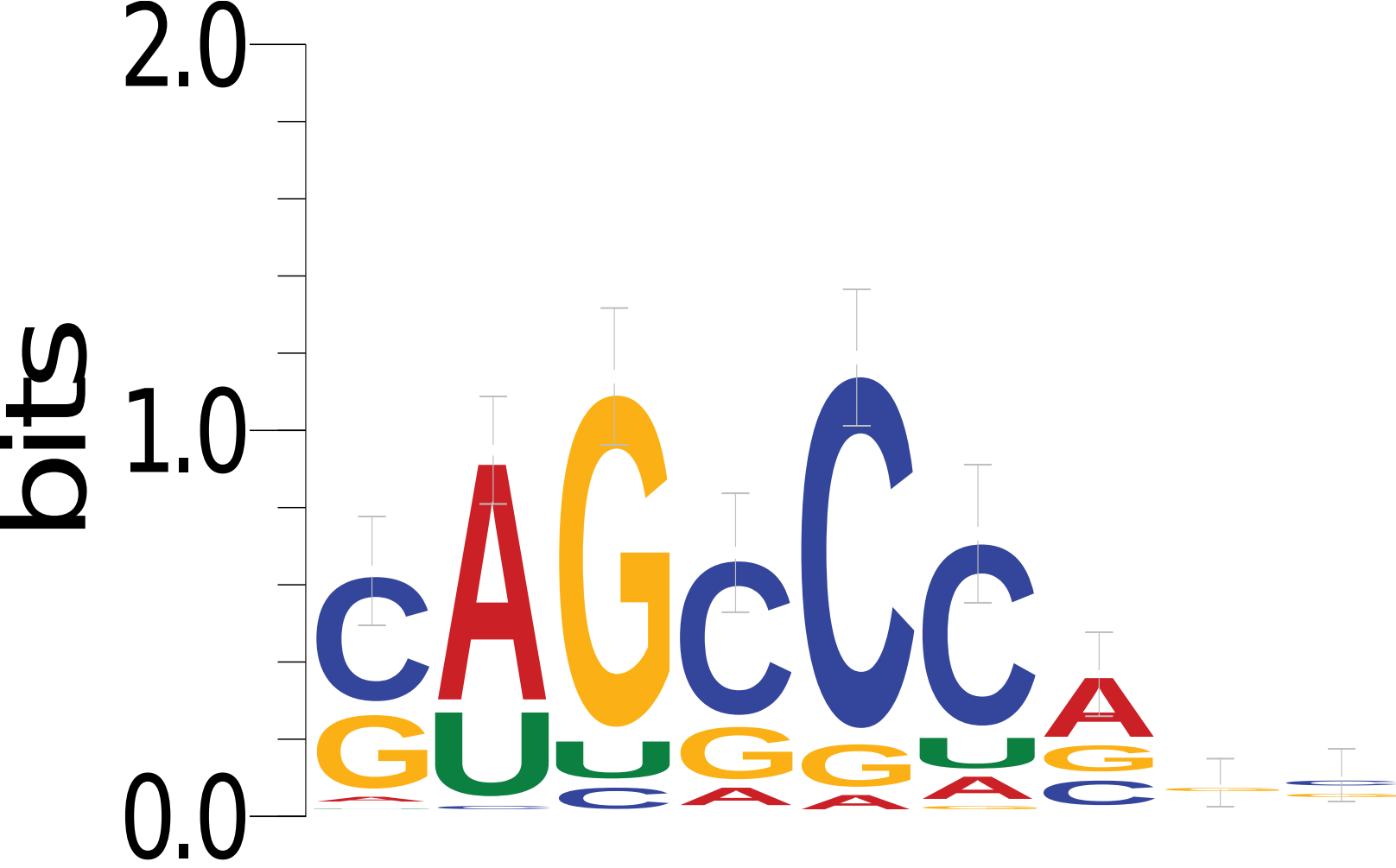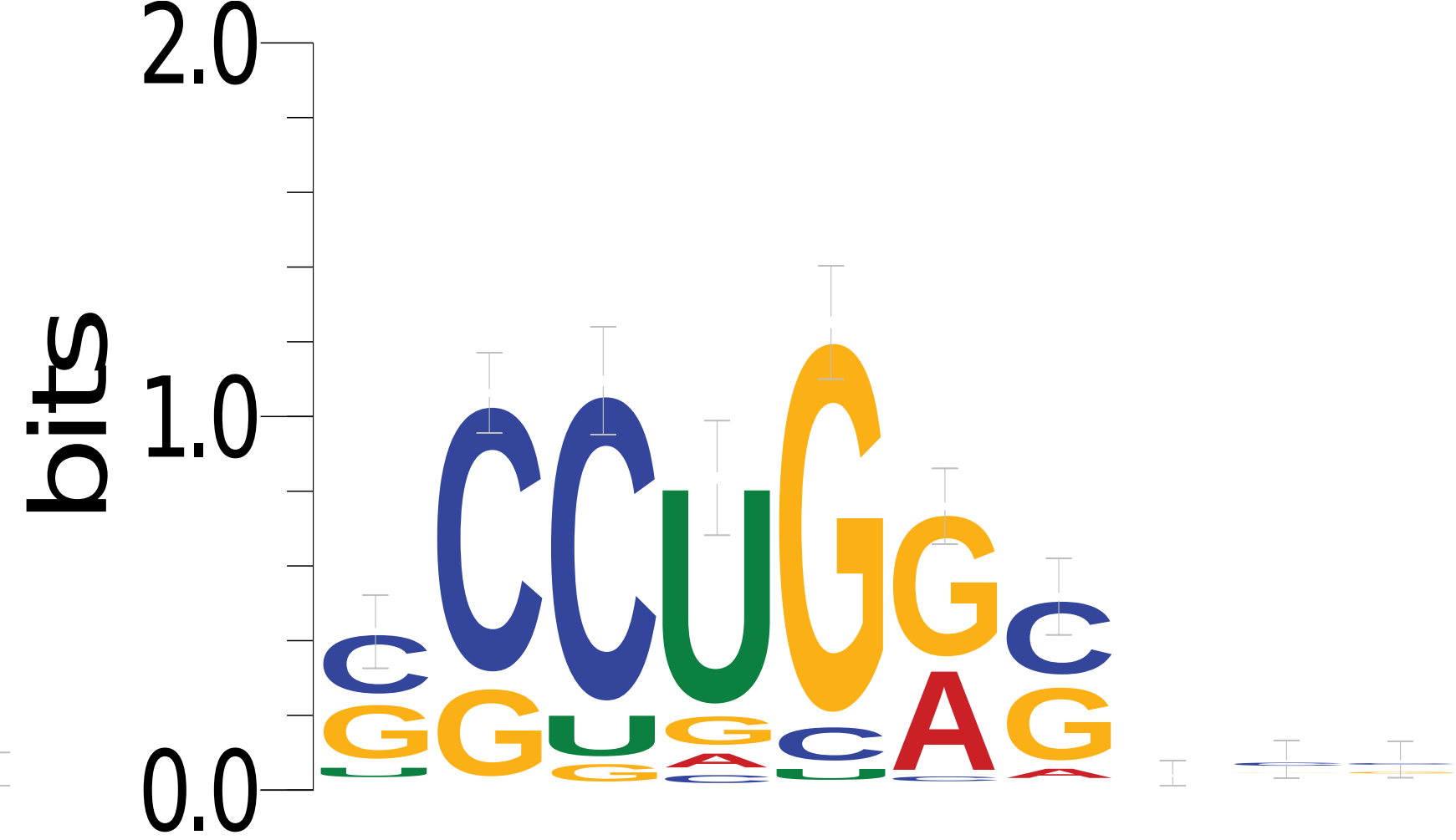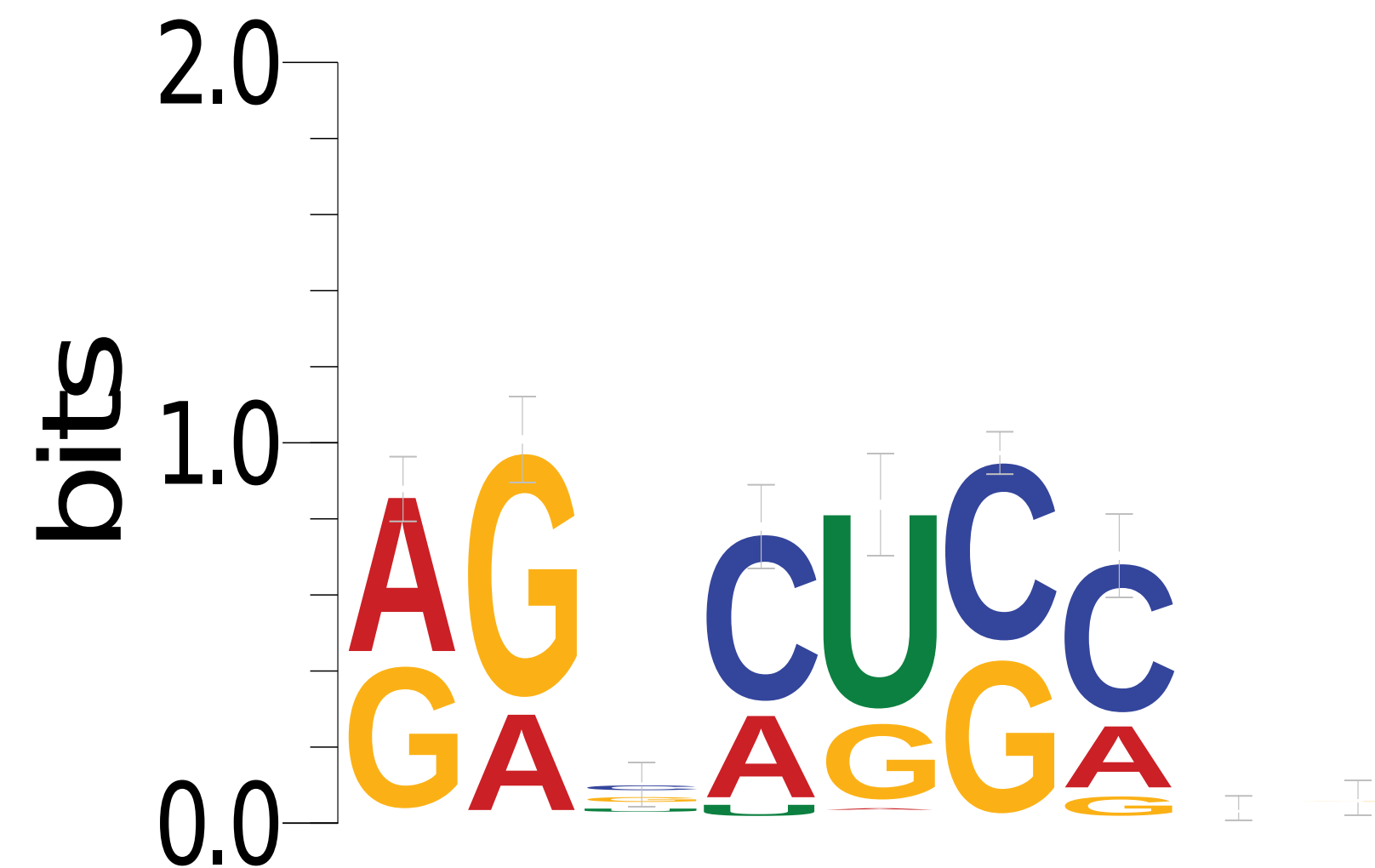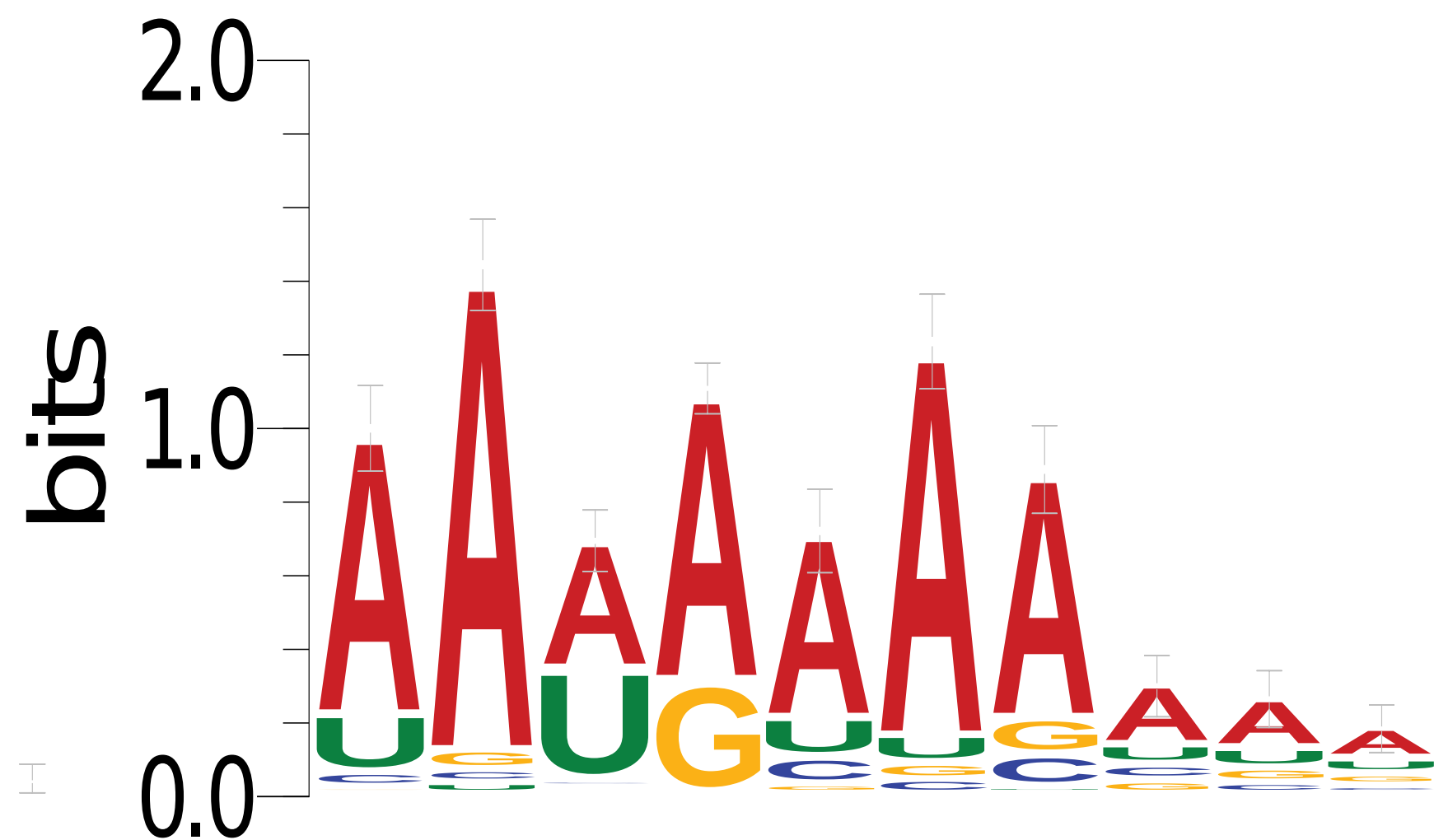

Supplement: Additional file 3 — Figure S2. The novel motifs still not verified by other studies are discoverd by iDeep. (PDF 65 kb) [file 12859_2017_1561_MOESM3_ESM.pdf]
