# Supplementary material for: RNA-protein binding motifs mining with a new hybrid deep learning based cross-domain knowledge integration approach
Source: BMC Bioinformatics. 2017 Feb 28;18:136. doi: 10.1186/s12859-017-1561-8 (PMC5331642; doi:10.1186/s12859-017-1561-8)
Supplement: Additional file 2 — Supplementary text and Table. Some details of iDeep. The principles about how to identify binding motifs by iDeep, the architecture of deep belief network and the discovered number of known motifs in CISBP-RNA. (PDF 127 kb) [file 12859_2017_1561_MOESM2_ESM.pdf]

Supplementary Materials

RNA-protein binding motifs mining with a new hybrid deep learning based cross-domain knowledge integration approach

Xiaoyong Pan and Hong-Bin Shen

1 Methods

1.1 Identifying binding motifs using iDeep

The CNN module in iDeep is able to detect binding motifs using its filters, which recognize the motif in a set of input sequences [1, 2]. For each sequence S_m and filter with size L, if the activation value A_{mfi} of filter f at position i is greater than the cut-off, e.g. $0.5\max_{mi} A_{mfi}$, then this sequence in windows L centring the position i is selected. After selecting the motif sequences, WebLogo is used for alignment and visualization.

$$A_{mfi} = ReLU(\sum_{l=1} \sum_{d=1}^{D} w_{fld} * s_{m,i+1,d}) \quad (1)$$

where $ReLU(x) = max(0, x)$, w_f is the weights of filter f, m is the sequence length. D is 4, the dimension of the one-hot encoding of nucleotide sequence.

1.2 Motif enrichment analysis

We use predicted motifs by iDeep to scan the input sequences, identify the binding sites. And then we do the same for the shuffled sequences as the background sequences. Here we applied the AME in the MEME suite [3] to get the motif enrichment.

1.3 The architecture of deep belief network

The architecture of DBN for input modalities clip-cobinding, Structure, Region type and Motif are as follows:
1. Fully connected layer
2. PReLU layer
3. BatchNormalization layer
4. Dropout layer
5. Fully connected layer
6. PReLU layer
7. BatchNormalization layer
8. Dropout layers
Additional layer for merging outputs from CNNs and DBNs:
9. Fully connected layer

10. Softmax layer

2 Results

Protein	# of motifs	protein	# of motifs	protein	# of motifs
1 Ago/EIF	8	12 ESWR1	14	22 Nsun2	15
2 Ago2M	13	13 FUS	11	23 PUM2	8
3 Ago2	13	14 Mut FUS	11	24 QKI	10
4 Ago2	15	15 IGF2.1-3	12	25 SRSF1	7
5 Ago2	8	16 hnRNPC	6	26 TAF15	12
6 eIF4AIII	8	17 hnRNPC	10	27 TDP-43	12
7 eIF4AIII	20	18 hnRNPL	11	28 TIA1	11
8 ELAVL1	15	19 hnRNPL	9	29 TIAL1	13
9 ELAVL1M	11	20 hnRNPL1	10	30 U2AF2	13
10 ELAVL1A	8	21 MOV10	13	31 U2AF2	10
11 ELAVL1	13				

Table S1: The discovered number of known motifs in CISBP-RNA agreeing with 102 filters from CNNs in iDeep for individual experiments.

References

[1] Alipanahi,B. *et al.* (2015) Predicting the sequence specificities of DNA- and RNA-binding proteins by deep learning. *Nat Biotechnol.* **33**, 831-8.

[2] Kelley,D.R. *et al.* (2016) Basset: learning the regulatory code of the accessible genome with deep convolutional neural networks. *Genome Res.* 3.

[3] Bailey TL, Johnson J, Grant CE, Noble WS. (2015) The MEME Suite. Nucleic Acids Res. 43(W1):W39-49. doi: 10.1093/nar/gkv416.
